# Hypochlorite-Modified LDL Induces Arrhythmia and Contractile Dysfunction in Cardiomyocytes

**DOI:** 10.3390/antiox11010025

**Published:** 2021-12-23

**Authors:** Chintan N. Koyani, Susanne Scheruebel, Ge Jin, Ewald Kolesnik, Klaus Zorn-Pauly, Heinrich Mächler, Gerald Hoefler, Dirk von Lewinski, Frank R. Heinzel, Brigitte Pelzmann, Ernst Malle

**Affiliations:** 1 Division of Molecular Biology and Biochemistry, Gottfried Schatz Research Center, Medical University of Graz, 8010 Graz, Austria; chini.koyani@novatium.at; 2 Department of Internal Medicine, Division of Cardiology, Medical University of Graz, 8036 Graz, Austria; gemason@wzhealth.com (G.J.); ewald.kolesnik@medunigraz.at (E.K.); dirk.von-lewinski@medunigraz.at (D.v.L.); 3 Division of Biophysics, Gottfried Schatz Research Center, Medical University of Graz, 8010 Graz, Austria; susanne.scheruebel@medunigraz.at (S.S.); klaus.zornpauly@medunigraz.at (K.Z.-P.); 4 The 2nd Affiliated Hospital and Yuying Children’s Hospital of Wenzhou Medical University, Wenzhou 325000, China; 5 Department of Surgery, Division of Cardiac Surgery, Medical University of Graz, 8036 Graz, Austria; heinrich.maechler@medunigraz.at; 6 Diagnostic and Research Center for Molecular BioMedicine, Diagnostic and Research Institute of Pathology, Medical University of Graz, 8010 Graz, Austria; gerald.hoefler@medunigraz.at; 7 Department of Internal Medicine and Cardiology, Charité-Universitätsmedizin Berlin, Campus Virchow-Klinikum, 13353 Berlin, Germany; Frank.heinzel@charite.de; 8 Deutsches Zentrum für Herz-Kreislauf-Forschung (German Centre for Cardiovascular Research), Partner Site Berlin, 10785 Berlin, Germany

**Keywords:** action potential, calcium transient, cardiomyocytes, cardiovascular disease, hypochlorite/hypochlorous acid, ion channel, low-density lipoprotein, MPO-H_2_O_2_-Cl- system, redox imbalance, scavenger receptor

## Abstract

Neutrophil-derived myeloperoxidase (MPO) and its potent oxidant, hypochlorous acid (HOCl), gained attention as important oxidative mediators in cardiac damage and dysfunction. As cardiomyocytes generate low-density lipoprotein (LDL)-like particles, we aimed to identify the footprints of proatherogenic HOCl-LDL, which adversely affects cellular signalling cascades in various cell types, in the human infarcted myocardium. We performed immunohistochemistry for MPO and HOCl-LDL in human myocardial tissue, investigated the impact of HOCl-LDL on electrophysiology and contractility in primary cardiomyocytes, and explored underlying mechanisms in HL-1 cardiomyocytes and human atrial appendages using immunoblot analysis, qPCR, and silencing experiments. HOCl-LDL reduced I_Ca,L_ and I_K1_, and increased I_NaL_, leading to altered action potential characteristics and arrhythmic events including early- and delayed-afterdepolarizations. HOCl-LDL altered the expression and function of CaV1.2, RyR2, NCX1, and SERCA2a, resulting in impaired contractility and Ca^2+^ homeostasis. Elevated superoxide anion levels and oxidation of CaMKII were mediated via LOX-1 signaling in HL-1 cardiomyocytes. Furthermore, HOCl-LDL-mediated alterations of cardiac contractility and electrophysiology, including arrhythmic events, were ameliorated by the CaMKII inhibitor KN93 and the I_NaL_ blocker, ranolazine. This study provides an explanatory framework for the detrimental effects of HOCl-LDL compared to native LDL and cardiac remodeling in patients with high MPO levels during the progression of cardiovascular disease.

## 1. Introduction

The prevalence of cardiovascular disease (CVD) is rising worldwide, making it the leading cause of deaths in developed countries. Understanding and investigating the exact etiology has been challenging due to the highly heterogeneous nature of CVD. Oxidative stress, inflammation, and immune cells play pivotal roles in the development and progression of CVD [1,2]. One of the most common first-line immune cell responses to cardiac inflammation and ischemia is neutrophil infiltration into the myocardium [3]. Neutrophils contain myeloperoxidase (MPO) and the enzyme, stored in azurophilic granules, is released into the extracellular space upon cell activation [4]. Clinical trials have correlated high circulatory MPO levels with the mortality in patients with coronary artery diseases, acute ischemic stroke, and heart failure (HF) [5]. Furthermore, high circulatory MPO levels are associated with an increased risk of atherosclerosis, ischemic heart disease, and myocardial infarction (MI) [6,7].

MPO catalyzes the reaction between H_2_O_2_ and Cl^−^ to generate hypochlorous acid/hypochlorite (HOCl/OCl^−^), a potent anti-bacterial molecule involved in the innate immune response. Therefore, the enzyme is considered as a front-line defender against microorganisms [3,8]. However, under chronic inflammatory conditions, MPO-derived HOCl is known to react with nucleic and amino acids as well as lipids and (lipo)proteins to generate oxidants that are detrimental to the host cells/tissues. One of the potential HOCl targets is apolipoprotein B-100 (apoB-100), the apolipoprotein moiety of low-density lipoprotein (LDL) [9]. While native LDL interacts with the classical LDL receptor, HOCl-modified LDL (HOCl-LDL) is known to interact with scavenger receptor-A1 and -B1 (SR-A1 and SR-B1), CD36, and lectin-like oxidized LDL receptor-1 (LOX-1) [10,11,12]. High levels of circulating LDL- and small-dense LDL-particles are linked to CVD [13], whereby oxidized/modified-LDL is considered to be a sensitive marker for the prediction of adverse cardiovascular events [14]. Most importantly, cardiomyocytes not only generate and secrete apoB-100 containing LDL-like particles [15,16], but also express the scavenger receptors [17]. Signaling events initiated by these receptors adversely affect cardiac function in CVD [17]. Based on these observations, it is plausible that the interaction of MPO-generated HOCl and cardiomyocyte-derived apoB-100 might adversely affect cardiomyocyte function.

Driven by this hypothesis, we aimed to establish a link between HOCl-LDL and the impairment of cardiac contractility and electrophysiology with a special focus on arrhythmic events. First, we used serial sections of infarcted and healthy human left ventricle (LV) to identify the presence of HOCl-modified epitopes and apoB-100. Next, we used primary guinea pig ventricular (GPV) cardiomyocytes to investigate the impact of HOCl-LDL on action potentials (APs), underlying ionic currents, Ca^2+^ homeostasis, and the expression patterns of candidate ion channels and receptors. In parallel, HL-1 cardiomyocytes were employed to unravel intracellular signaling cascades. Finally, we used pharmacological interventions to ameliorate the detrimental effects of HOCl-LDL on cardiomyocyte function.

## 2. Materials and Methods

### 2.1. Isolation and Modification of Native LDL

Native LDL (d = 1.035–1.065 g/mL) was isolated from the plasma of healthy normolipidemic volunteers by ultracentrifugation as described previously [18]. The protein content of the final LDL preparation consisted of 96–98% apoB-100, as measured immunochemically [18]. Modification of LDL by NaOCl (HOCl-LDL) at concentrations of 0.4 or 0.8 mM (in the absence of free amino acids/carbohydrates) resulted in an oxidant:lipoprotein molar ratio of 200:1 or 400:1, respectively [11]. The reaction mixture was kept at 4 °C overnight. Modification of native LDL by the MPO-H_2_O_2_-Cl^−^ system (termed MPO-LDL) was performed as described previously [19]. Briefly, native LDL (500 µg/mL in PBS, pH 7.4) was kept at 37 °C for 10 min, H_2_O_2_ was added 20 times at 5 min intervals to a final concentration of 20 μM. Along with the first and then every third H_2_O_2_ addition step, 1 μg MPO (Planta Natural Products, 1120 Vienna, Austria) was added, resulting in 8 additions of MPO. The reaction mixture was kept at 37 °C for 3 h in total and further at 4 °C overnight. All modified LDL preparations were filtered through a PD10 column immediately before use.

### 2.2. Cell Culture

The mouse atrial cardiomyocyte cell line (HL-1 cardiomyocytes) was cultured in fibronectin (0.5%, *w*/*v*)/gelatin (0.02%, *w*/*v*) (Sigma-Aldrich, 1060 Vienna, Austria) coated flasks and supplied with Claycomb medium containing 10% (*v*/*v*) fetal bovine serum, 0.1 mM norepinephrine, 2 mM L-glutamine, 100 IU/mL penicillin, and 100 µg/mL streptomycin (Sigma-Aldrich) [20]. Cardiomyocytes were used between passage 60–80 and maintained at 37 °C under 5% CO_2_.

### 2.3. Isolation of Primary GPV Cardiomyocytes

Cardiomyocytes were isolated from adult guinea pigs (GPs, Dunkin-Hartley of either sex, Charles River Laboratories, 97633 Sulzfeld, Germany). The experimental procedure and the number of used animals were approved by the ethics committee of the Federal Ministry of Science, Research and Economy of the Republic of Austria (BMWF-66.010/0110-WF/V/3b/2016). GPs were euthanized, the hearts were quickly removed, and cardiomyocytes were isolated as described previously [21] using collagenase 2 (Worthington Biochemical Corporation [Lakewood, NJ, 08701, USA], 100 IU/mL in a buffer [composition in mM: NaCl 126, KCl 4.7, KH_2_PO_4_ 1.2, MgSO_4_ 2.5, NaHCO_3_ 2.49, HEPES/Na^+^ 0.5, CaCl_2_ 0.025, and D(+)-glucose 5.6, pH 7.4 adjusted with NaOH]). After enzymatic digestion and raising the Ca^2+^ concentration to 1 mM, cardiomyocytes were transferred to the cell culture medium M199 (Sigma-Aldrich) containing 50 IU/mL penicillin and 50 µg/mL streptomycin (Sigma-Aldrich), and maintained at 37 °C under 5% CO_2_. All experiments were performed on the day after isolation. Prior to electrophysiological recordings and Ca^2+^ transient (CaT) experiments, cardiomyocytes were maintained in suspension, which settled onto glass coverslips within a few seconds immediately before the experiments. For quantitative real-time PCR (qPCR) analysis, cardiomyocytes were transferred to plates coated with 5 µg/mL laminin and 20 µg/mL L-ornithine to separate viable cardiomyocytes from the dead ones.

### 2.4. Electrophysiological Recordings and Analysis

Ionic currents and APs were recorded in the whole cell configuration of the patch clamp technique using an Axopatch 200B amplifier (Molecular Devices [San Jose, CA, 95134, USA]) and the A/D-D/A converter Digidata 1322A (Molecular Devices). Cell membrane capacitance was determined by the integration of the capacitive transient elicited by a 10 mV hyperpolarizing step from −50 mV and ion currents were normalized to cell membrane capacitance, and expressed as pA/pF to compensate for cell size variations. In order to allow for equilibration of the pipette solution with the cytosol, current recordings were started 4 min after the rupture of the membrane patch. Late Na^+^ current (I_NaL_) was measured at 23 ± 1 °C, while all other currents and APs were recorded at 37 ± 1 °C.

To record APs, cardiomyocytes were superfused with normal Tyrode’s (NT) solution (composition in mM: NaCl 137, KCl 5.4, CaCl_2_ 1.8, MgCl_2_ 1.1, NaHCO_3_ 2.2, NaH_2_PO_4_ 0.4, HEPES/Na^+^ 10, and D(+)-glucose 5.6, pH 7.4 was adjusted with NaOH) and pipettes were filled with an internal solution (containing in mM: KCl 110, ATP/K^+^ 4.3, MgCl_2_ 2, CaCl_2_ 1, EGTA 11, and HEPES/K^+^ 10, pH 7.4 adjusted with KOH, estimated free [Ca^2+^] < 10^−8^ M). For AP recordings, cardiomyocytes were stimulated with minimal suprathreshold current pulses (5 ms) at a 0.5 or 1 Hz frequency. In order to exclude any initial transient behavior, the first 10 APs were excluded from the analysis. The following 10 consecutive APs were analyzed using MATLAB software [22].

Ba^2+^ (0.5 mM) was added to the NT solution in order to measure inward rectifier potassium current (I_K1_) as a Ba^2+^-sensitive current. I_K1_ was elicited by hyperpolarizing voltage steps (3 s) from −40 mV to −130 mV (10 mV increments, holding potential −40 mV, see respective Figure inset). Currents (at the end of the pulse) measured in the presence of BaCl_2_ were subtracted from the currents in the absence of BaCl_2_ for the same myocyte [23].

For studying L-type Ca^2+^ current (I_Ca,L_), KCl was replaced by equimolar CsCl in both, the external and the internal solutions. I_Ca,L_ was elicited by voltage steps to potentials between −40 and +90 mV (10 mV interval, 400 ms, see respective Figure inset), preceded by a 100 ms pre-pulse to −40 mV from a holding potential of −80 mV (in order to activate and voltage-inactivate sodium current). The amplitude of I_Ca,L_ was measured as the difference between the peak inward current and the current at the end of the depolarization pulse [21]. The reversal potential of I_Ca,L_ did not differ between the control and treated groups and the mean reversal potential of all measured myocytes was +54 mV. For the determination of steady-state activation of I_Ca,L_, peak values of I_Ca,L_ were divided by the driving force and normalized with the maximal value. Curves were fitted to the normalized data according to the equation.

$$d_{\infty }=1/\left\{1+exp\left(V_{1/2\;act}-V\right)/k\right\}$$

where *d* is the Boltzmann function, *V* is the membrane potential, *V*_1/2_ *_act_* is the membrane potential of half-activation, and *k* is the slope of the activation curve.

To determine steady-state inactivation of I_CaL_, current was activated with test pulses to +10 mV (400 ms), which were preceded by conditioning pulses from −45 to +50 mV (5 mV interval, 400 ms) from a holding potential of −45 mV (Supplementary Figure S2C, inset). The pulse pair was separated by a short 10 ms repolarizing step to −45 mV. I_Ca,L_ during the test step was normalized and plotted as a function of pre-pulse potential. Curves were fitted to the normalized data according to the equation.

$$f_{\infty }=1/\left\{1+exp\left(V-V_{1/2\;inact}\right)/k\right\}$$

where *f* is the Boltzmann function, *V* is the membrane potential, *V*_1/2_ *_inact_* is the membrane potential of half-inactivation, and *k* is the slope of the inactivation curve.

I_NaL_ was measured using an extracellular solution containing (in mM) NaCl 130, tetraethylammonium chloride 10, CsCl 4, MgCl_2_ 1, D(+)-glucose 10, and HEPES 10, pH 7.4 was adjusted with NaOH, and an internal solution containing (all given in mM) CsCl 40, Cs-glutamate 80, NaCl 5, MgCl_2_ 0.92, Mg-ATP 5, Li-GTP 0.3, HEPES 10, niflumic acid 0.03, nifedipine 0.02, and strophanthidin 0.004, pH 7.4 was adjusted with CsOH (estimated free [Ca^2+^] < 10^−8^ M). I_NaL_ was determined by a train of voltage pulses (5 pulses of each 1000 ms, basic cycle length 2 s) to −20 mV from a holding potential of −120 mV (see respective Figure inset). Each pulse was preceded by a 5 ms pre-pulse to +50 mV to optimize the voltage control [24]. The time course of current inactivation was fitted bi-exponentially and the slow time constant (τ_slow_) largely corresponding to I_NaL_ was analyzed [25].

### 2.5. Incubation of Right Atrial Appendages (RAAs) with HOCl-LDL

Human RAAs were obtained as excess tissue from 3 patients undergoing cardiac surgery. The use of human tissue was approved by the ethics committee of the Medical University of Graz, Austria, and all patients gave informed consent. All experiments were carried out in accordance with the Declaration of Helsinki. Tissues were provided by the Division of Cardiology, Medical University of Graz, Austria, and transported in a cold NT solution containing 5 mM 2,3-butanedione 2-monoxime (Sigma-Aldrich) and 500 µM CaCl_2_ to the laboratory. Tissues were cut into small pieces (~1 mm^3^) and transferred to NT containing LDL or HOCl-LDL (oxidant:lipoprotein ratio of 200:1, 250 µg/mL) and incubated for 8 h with oxygen supply (37 °C). Afterward, tissues were washed with cold PBS and used for qPCR and immunoblot analysis.

### 2.6. Immunoblot Experiments

Cardiomyocytes and tissues were lysed/homogenized in ice-cold lysis buffer (50 mM HEPES, 150 mM NaCl, 1 mM EDTA, 10 mM Na_4_P_2_O_7_, 2 mM Na_3_VO_4_, 10 mM NaF, 1% [*v*/*v*] Triton X-100, and 10% [*v*/*v*] glycerol, pH 7.4) containing a protease/phosphatase inhibitor cocktail (Thermo Scientific, 1200 Vienna, Austria) for 10 min on ice. After protein estimation, 50 µg of total protein content was added to 10 µL of 4x NuP AGE LDS sample buffer (Invitrogen, 5090 Lofer, Austria) containing 2 µL sample reducing agent (Invitrogen) and heated (70 °C, 10 min). Proteins were separated by electrophoresis on NuPAGE 4–12% Bis-Tris gels and transferred to nitrocellulose membranes (0.2 µM, Invitrogen) [22]. Membranes were blocked with 5% (*w*/*v*) non-fat milk in a Tris-buffered saline containing Tween 20 (TBST, 25 °C, 2 h) and incubated with anti-oxidized Ca^2+^/calmodulin-dependent protein kinase II (oxCaMKII, M^281/282^, 1:1000, Millipore-07-1387 [Millipore, 1147 Vienna, Austria]) primary antibody (diluted in 5% [*w*/*v*] bovine serum albumin (BSA)-TBST) overnight at 4 °C. Membranes were washed and incubated with horseradish peroxidase-conjugated goat anti-rabbit IgG (1:200,000 Biomol-6293 [Biomol GmbH, 22525 Hamburg, Germany], 25 °C, 2 h). Immunoreactive bands were visualized using Super Signal West Pico Chemiluminescent substrate (Thermo Scientific) and the Bio-Rad ChemiDoc MP Imaging System (Bio-Rad, 1130 Vienna, Austria). For normalization, membranes were stripped with stripping buffer (58.4 g/L NaCl, 7.5 g/L glycine, pH 2.15) and incubated with anti-CaMKII (1:1000 Santa Cruz-SC-9035 [Santa Cruz Biotechnology, Inc., Dallas, TX, 75220, USA]) as the primary antibody.

### 2.7. qPCR

Total RNA was isolated from cardiomyocytes or tissues using a Direct-zol RNA MiniPrep kit (Zymo Research, 3032 Eichgraben, Austria). One µg of RNA was subjected to reverse transcription. Six ng of cDNA per template was used for gene quantification using a GoTaq qPCR Master Mix (Promega, 1060 Vienna, Austria) and gene specific primers (see Table 1). The qPCR protocol was performed using the LightCycler 480 system (Roche Diagnostics, 1210 Vienna, Austria) [26]. Relative gene expression levels compared to GAPDH were calculated using ΔΔCT method.

### 2.8. Measurement of Reactive Oxygen Species (ROS) and Reactive Nitrogen Species (RNS)

Intracellular ROS/RNS levels were assessed using 5-(and -6)-carboxy-2′,7′ dichlorodihydrofluorescein diacetate (carboxy-H2DCFDA, Invitrogen), a cell-permeable dye that becomes fluorescent upon oxidation by ROS/RNS. After treatment, HL-1 cardiomyocytes were incubated with 10 μM DCFDA in PBS for 30 min at 37 °C. Afterward, cardiomyocytes were washed twice with ice-cold PBS and lysed with 300 μL of 3% (*v*/*v*) Triton X-100 in PBS (30 min) followed by the addition of 50 µL absolute ethanol (15 min) with shaking (1350 rpm, 4 °C). The supernatant was used to measure DCF fluorescence at emission and correction wavelengths of 485 and 540 nm, respectively [26].

### 2.9. Scavenger Receptor Silencing by siRNA

HL-1 cardiomyocytes were transfected with four different siRNAs specific for SR-A1, SR-B1, CD36, or LOX-1 (40 nM, SI04945962, SI02672971, SI00945063, or SI02676765, respectively, Qiagen, 40724 Hilden, Germany), or with a scrambled negative control siRNA (40 nM, 1022076, Qiagen). The siRNA transfections were performed using Lipofectamine 3000 (Invitrogen) according to the manufacturer’s suggestions [22]. Cardiomyocytes were used 48 h after the transfection for further experiments.

### 2.10. Cell Shortening and CaT Measurements

After treatment, cardiomyocytes were washed twice with NT solution and incubated with NT solution containing 1 µM Fura-2-AM and 1 µM Pluronic F-127 (Thermo Fisher) for 30 min at 25 °C. CaT was assessed by field stimulation (platinum electrode distance: 1 cm; pulse duration: 5 ms; suprathreshold pulse amplitude: 4 V/cm) at a 1 Hz frequency using a video-based cell length detection system (IonOptix Corporation [Westwood, MA, 02090, USA]) at 37 °C. Fluorescence intensities were measured at 340 and 380 nm of excitation and at 510 nm of emission wavelengths using a dual excitation light source. The F340/F380 ratio was used as an index of cytosolic Ca^2+^ concentration and to calculate CaT relaxation tau (τ). In parallel, cardiomyocytes were rapidly superfused with 30 mM caffeine without electrical stimulus in order to assess NCX1 function. Data were analyzed using Clampfit 10.2 (Molecular Devices [San Jose, CA, 95134, USA) and LabChart 7.0 (peak analysis module, ADInstruments Ltd. [Oxford, OX4 6HD, U.K.]) [22].

### 2.11. Immunohistochemistry

Formalin-fixed, paraffin-embedded serial sections (3 µm) of non-infarcted and infarcted (infarcted scar and border zone) LV of healthy and MI patients were obtained from the Diagnostic and Research Institute of Pathology, Medical University of Graz, Austria. The use of samples was approved by the ethics committee of the Medical University of Graz, Austria (28-097ex15/16). Sections were deparaffinized in xylene and rehydrated in ethanol stepwise, with the gradual decrease in % of ethanol. Sections were blocked with endogenous peroxidase (3% [*v*/*v*] H2O2 in methanol, 15 min) and incubated for 1 h at 25 °C with the following primary antibodies (diluted in Dako REAL Antibody Diluent, Dako, 1190 Vienna, Austria): anti-CD66 (1:10, Novocastra-NCL-CD66a [Novocastra-Leica Biosystems, 1170 Vienna]), anti-MPO (1:400, Thermo Scientific-RB-373-A1), anti-apoB-100 (1:50, [27]), anti-HOCl-modified epitopes (1:1, [28]), anti-CaMKII (1:30, Santa Cruz-SC-9035), or anti-oxCaMKII (1:50, Millipore-07-1387). After washing three times with PBS, sections were incubated for 30 min at 25 °C with UltraVision LP Large Volume HRP Polymer (Thermo Scientific) to visualize the reaction. Detection was performed using AEC Substrate Chromogen (Dako, 1190 Vienna, Austria, 5 min). Counterstaining was performed with hematoxylin (60 s). Negative staining was performed in the absence of primary antibodies [29].

### 2.12. Statistical Analysis

Statistical analyses were performed using IBM SPSS Statistics 26 software. The approximate normal distribution of data was assessed by visual (histograms and normal Q-Q plots) and numerical investigation (z-value of skewness and kurtosis; *p* value of Shapiro–Wilk test). After checking the homogeneity of variance by Levene’s test, between groups comparisons were evaluated by unpaired Student’s t-test or one-way ANOVA (followed by Tukey’s post-hoc test). A *p*-value < 0.05 was considered statistically significant. All tests were 2-sided.

## 3. Results

### 3.1. Neutrophils, MPO, apoB-100, and HOCl-Modified Epitopes Accumulate in the Infarcted Myocardium

**Figure 1:**
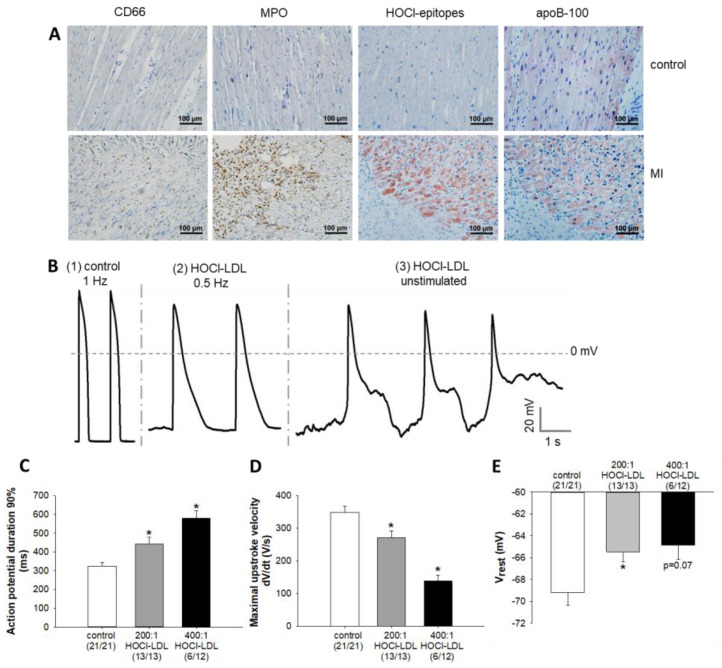
Immunostaining of neutrophils, MPO, HOCl-epitopes, and apoB-100 in LV tissue, and the modulation of cardiac AP parameters by HOCl-LDL. (**A**) Representative immunostainings of neutrophils (CD66, brown dots), MPO (brown dots), HOCl-epitopes (red), and apoB-100 (red) in the serial sections of LV from healthy volunteers (control, upper panels) and patients with myocardial infarction (MI, lower panels). Cardiomyocyte nuclei were stained blue with hematoxylin. (**B**) Representative APs of (1) control, and (2,3) HOCl-LDL-incubated (oxidant:lipoprotein molar ratio of 400:1, 250 µg/mL, 12–16 h) GPV cardiomyocytes that were stimulated at a frequency of (1) 1 Hz, (2) 0.5 Hz, or (3) unstimulated. (**C**) APD at 90% repolarization, (**D**) maximal upstroke velocity, and (**E**) resting membrane potential (V_rest_) of GPV cardiomyocytes incubated with HOCl-LDL (oxidant:lipoprotein molar ratio of 200:1 or 400:1, 250 µg/mL, 12–16 h, 1 Hz stimulation frequency). Values are expressed as mean ± SEM. (*n/n*) represents the number of myocytes showing stimulated APs/total number of patched myocytes. Cardiomyocytes showing arrhythmic events were excluded from the analysis. * indicates *p* < 0.05 vs. control.

To examine in vivo relevance of neutrophils, MPO, HOCl-epitopes, and LDL (apoB-100), we performed immunohistochemistry using serial sections of infarcted and healthy (control) human LV. A pronounced staining of CD66-positive cells, a marker for neutrophils, became apparent in the infarcted scar region (Figure 1A). Concomitantly, pronounced MPO staining was found not only in the infarcted scar extracellular matrix, but also associated with some neutrophils (Figure 1A). Thus, neutrophil infiltration and activation paralleled the increased MPO expression in the infarcted myocardium. Using a specific monoclonal antibody [28], we observed abundant staining of HOCl-modified epitopes associated with cardiomyocytes in the infarcted border regions, indicating that the MPO-H_2_O_2_-Cl^−^ system is active in the infarcted myocardium (Figure 1A). On the contrary, an insignificant staining of CD66 and MPO was observed in healthy myocardium, whereas HOCl-modified epitopes could not be detected, revealing the absence of MPO activity.

Previous studies have reported the production and secretion of apoB-100-containing lipoproteins by cardiomyocytes [15,16]. Similarly, an indistinguishable staining pattern of apoB-100 became apparent between both the groups, whereby the staining was mainly localized to cardiomyocytes. Interestingly, the staining patterns of apoB-100 matched to those of HOCl-modified epitopes in the infarcted border regions. This observation, along with the fact that the monoclonal antibody used for the detection of HOCl-modified epitopes was raised against HOCl-LDL (modified in vitro [28]) and recognizing HOCl-modified apoB-100 in human lesion material [27], supports an in vivo relevance of LDL modification by HOCl.

### 3.2. Alteration of Action Potential Parameters in Response to HOCl-LDL

Elevated levels of myocardial MPO and its oxidants are considered to impair cardiac electrophysiology [30,31]. Therefore, to investigate the functional impact of the interaction of HOCl-LDL with cardiomyocytes, we analyzed AP characteristics. Figure 1B(1) displays representative APs of a control GPV cardiomyocyte (1 Hz stimulation frequency). Next, we used two different HOCl concentrations to modify native LDL. Deleterious alterations in AP characteristics became apparent in response to HOCl-LDL. Figure 1B(2) demonstrates a prolongation of AP duration (APD) and depolarization of resting membrane potential (V_rest_) of a 400:1 HOCl-LDL-incubated cardiomyocyte stimulated at a lower frequency (0.5 Hz, due to prolonged APD). Approximately 50% of cardiomyocytes incubated with HOCl-LDL (400:1 compared to 200:1) showed arrhythmias, including early- and delayed- afterdepolarizations (EADs and DADs, Figure 1B(3)).

For a statistical comparison of AP parameters, only non-arrhythmic cardiomyocytes stimulated at 1 Hz frequency were used. A significant APD prolongation at 90% repolarization (Figure 1C) became evident (325 ± 17 ms [control] vs. 442 ± 35 ms [HOCl-LDL, 200:1] and 579 ± 37 ms [HOCl-LDL, 400:1]). Furthermore, HOCl-LDL treatment significantly reduced the maximal upstroke velocity of APs (348 ± 19 V/s [control] vs. 271 ± 21 V/s [HOCl-LDL, 200:1] and 138 ± 17 V/s [HOCl-LDL, 400:1], Figure 1D) and depolarized resting membrane potential V_rest_ (−69.2 ± 1.1 mV [control] vs. −65.5 ± 0.9 mV [HOCl-LDL, 200:1], Figure 1E). Taken together, HOCl-LDL impairs cardiac cellular excitability and induces arrhythmias.

### 3.3. HOCl-LDL Raises Intracellular Superoxide Levels and, in turn, Oxidizes CaMKII via LOX-1 Signaling

**Figure 2:**
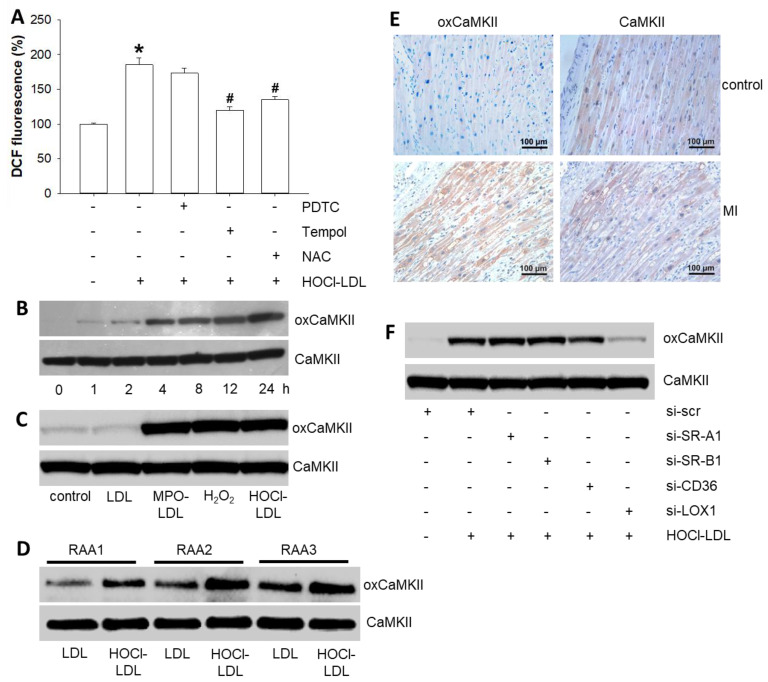
Induction of superoxide anions and LOX-1-mediated CaMKII oxidation by HOCl-LDL. (**A**) DCF fluorescence intensities in HL-1 cardiomyocytes incubated with HOCl-LDL (oxidant:lipoprotein molar ratio of 200:1, 250 µg/mL, 1 h) with or without ROS/RNS scavengers (PDTC [1 mM], Tempol [1 mM], or NAC [5 mM], 30 min pre-treatment) (*n* = 6). (**B**) Time-dependency of CaMKII oxidation in HL-1 cardiomyocytes treated with HOCl-LDL (oxidant:lipoprotein molar ratio of 200:1, 250 µg/mL) for indicated time periods (*n* = 6). (**C**) CaMKII oxidation in HL-1 cardiomyocytes incubated with native LDL (250 µg/mL, 8 h), MPO-LDL (250 µg/mL, 8 h), H_2_O_2_ (100 µM, 1 h), or HOCl-LDL (oxidant:lipoprotein molar ratio of 200:1, 250 µg/mL, 8 h) (*n* = 6). (**D**) oxCaMKII expression in three human RAAs incubated with either native LDL or HOCl-LDL (oxidant:lipoprotein molar ratio of 200:1, 250 µg/mL, 8 h) (*n* = 3). I Representative immunostainings of oxCaMKII (red) and CaMKII (red) in healthy (control, upper panels) and infarcted border regions (MI, lower panels) of the myocardium. Cardiomyocyte nuclei were stained blue with hematoxylin. (**F**) HOCl-LDL (oxidant:lipoprotein molar ratio of 200:1, 250 µg/mL, 8 h)-induced oxCaMKII expression in HL-1 cardiomyocytes transfected with scrambled siRNA (si-scr) (40 nM) or siRNA against SR-A1 (si-SR-A1), SR-B1 (si-SR-B1), CD36 (si-CD36), or LOX-1 (si-LOX-1) (40 nM) (*n* = 6). Values are expressed as mean ± SEM. (*n*) represents the number of experiments. * indicates *p* < 0.05 vs. control and # indicates *p* < 0.05 vs. HOCl-LDL.

In search of the underlying mechanism(s) of HOCl-LDL-mediated effects on cardiomyocyte Aps, we focused on intracellular redox status, as HOCl-LDL has been reported to elevate ROS/RNS levels [32]. A time-dependent increase in intracellular ROS/RNS levels became apparent starting from 15 min in HL-1 cardiomyocytes incubated with HOCl-LDL (Supplementary Figure S1A). To narrow down on the specific type of reactive species, blockers of superoxide and/or nitric oxide anions were used. We observed a significant reduction in elevated ROS/RNS levels in response to Tempol (a specific scavenger of superoxide anion) and N-acetylcysteine (NAC, a mixed scavenger of nitric oxide and superoxide anions, Figure 2A).

On the contrary, a specific scavenger of nitric oxide anion, pyrrolidine dithiocarbamate (PDTC), failed to blunt HOCl-LDL-induced DCF fluorescence. Thus, we concluded that the superoxide anion is the major reactive species elevated in cardiomyocytes when stimulated with HOCl-LDL.

Superoxide anions primarily undergo dismutation to generate H_2_O_2_, a key player in the oxidation of various proteins. In fact, H_2_O_2_ targets one of the most crucial multifunctional cardiac proteins, CaMKII [33], that regulates several ion channels, pumps, and proteins, thereby playing a key role in cardiac excitability and Ca^2+^ homeostasis [34]. Hence, we performed immunoblots to follow CaMKII oxidation at the M^281/282^ residue. In response to HOCl-LDL treatment, we observed a time-dependent oxidation of CaMKII in HL-1 cardiomyocytes (Figure 2B) that became apparent starting after 4 h. In contrast, HOCl-LDL treatment did not alter the phosphorylation of CaMKII in HL-1 cells (data not shown). HOCl-LDL-induced CaMKII oxidation was corroborated by the levels of CaMKII oxidation in response to (i) native LDL (a negative control), (ii) LDL modified by the MPO-H_2_O_2_-Cl^−^ system (MPO-LDL), and (iii) H_2_O_2_ (a positive control) (Figure 2C). To validate these findings further, human RAAs were cut into 1 mm^3^ pieces, divided into two groups, and incubated with either native LDL or HOCl-LDL. Compared to native LDL, HOCl-LDL-incubated RAAs from all three patients showed elevated CaMKII oxidation (Figure 2D). Additionally, immunohistochemistry data revealed pronounced oxCaMKII expression in the infarcted border zones, but not in healthy human myocardium (Figure 2E).

Modified lipoproteins are known ligands/agonists for SR-A1, SR-B1, CD36, and LOX-1 [10]. Based on results mentioned above, we aimed to identify the candidate target receptor for HOCl-LDL promoting CaMKII oxidation. In response to siRNA transfection of HL-1 cardiomyocytes, mRNA expression levels of these receptors were reduced significantly (Supplementary Figure S1B). HOCl-LDL treatment could oxidize CaMKII in SR-A1-, SR-B1-, and CD36-silenced cardiomyocytes to a similar extent to that of the controls (si-scr); however, it failed to do so in LOX-1-silenced cardiomyocytes (Figure 2F). These data suggest LOX-1 as a likely target receptor for HOCl-LDL and CaMKII oxidation, respectively (densitometric evaluation of immunoreactive bands is shown in Supplementary Figure S3). In summary, the interaction of HOCl-LDL with LOX-1, and the production of superoxide anions, oxidize CaMKII in cardiomyocytes.

### 3.4. HOCl-LDL Modulates the Expression and Function of Ion Channels via CaMKII Oxidation

**Figure 3:**
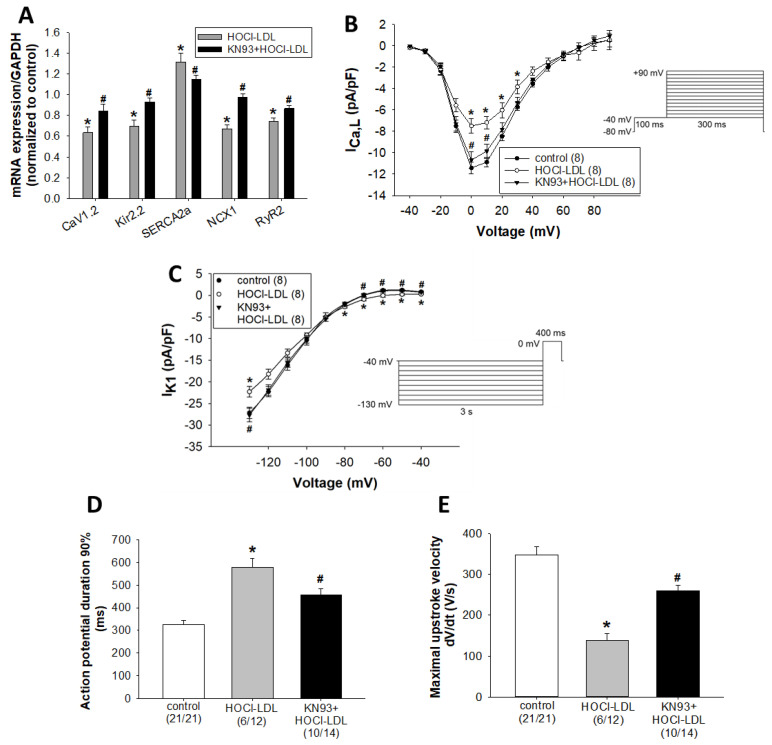
HOCl-LDL modulates the expression of ion channels/pumps, and reduces I_Ca,L_ as well as I_K1_ densities via CaMKII oxidation. (**A**) mRNA expression of indicated ion channels/pump in HL-1 cardiomyocytes incubated with HOCl-LDL (oxidant:lipoprotein molar ratio of 200:1, 250 µg/mL, 12 h) in the absence or presence of KN93 (5 µM, added 30 min prior to HOCl-LDL) (*n* = 6). (**B**) I_Ca,L_ and (**C**) I_K1_ density (the voltage clamp protocols are illustrated in the respective insets), (**D**) APD at 90% repolarization, and € maximal upstroke velocity of GPV cardiomyocytes incubated with (B,C) HOCl-LDL (oxidant:lipoprotein molar ratio of 200:1) or (**D**,**E**) HOCl (400:1, 250 µg/mL, 12–16 h, 1 Hz stimulation frequency) in the absence or presence of KN93 (5 µM, added 30 min prior to addition of HOCl-LDL). Values are expressed as mean ± SEM. (*n*) represents the number of (**A**) experiments or (**B**,**C**) cardiomyocytes, and (**D**,**E**) (n/n) represents the number of myocytes showing stimulated APs/total number of patched myocytes. Cardiomyocytes showing arrhythmic events were excluded from the analysis. * indicates *p* < 0.05 vs. control, # indicates *p* < 0.05 vs. HOCl-LDL.

Next, we investigated whether CaMKII oxidation contributes to the observed electrophysiological disturbances induced by HOCl-LDL. In this regard, we performed expression, as well as functional, experiments. Out of the tested ion channels and pumps, we observed a reduced mRNA expression of the alpha 1C subunit of the voltage-gated L-type Ca^2+^ channel (CaV1.2), inward rectifier voltage-gated K^+^ channel (Kir2.2), sodium-calcium exchanger 1 (NCX1), and ryanodine receptor 2 (RyR2), but an increased sarcoplasmic reticulum Ca^2+^-ATPase 2a (SERCA2a) mRNA expression in HL-1 cardiomyocytes treated with HOCl-LDL (Figure 3A). Similar effects were also detected in GPV cardiomyocytes (Supplementary Figure S1C) and human RAAs (Supplementary Figure S1D). Interestingly, mRNA expression of Kir2.1 and Kir2.3 was unchanged in GPV cardiomyocytes (Supplementary Figure S1C) and human RAAs (Supplementary Figure S1D). In addition, the pre-treatment of HL-1 cardiomyocytes with KN93 reversed the HOCl-LDL-induced alterations in mRNA expression of the ion channels and pumps (Figure 3A), suggesting a role of oxCaMKII in this process.

The reduced CaV1.2 expression led us to measure I_Ca,L_ density and its voltage-dependence of steady-state activation and inactivation in GPV cardiomyocytes. In contrast to the unresponsiveness to native LDL (Supplementary Figure S2A), HOCl-LDL significantly reduced I_Ca,L_ density at membrane potentials between 0 mV and +30 mV (Figure 3B). The maximum I_Ca,L_ density was observed at 0 mV membrane potential (11.42 ± 0.54 pA/pF [controls] vs. 7.49 ± 0.67 pA/pF [HOCl-LDL]). In contrast to the current density, HOCl-LDL altered neither steady-state activation nor inactivation parameters of I_Ca,L_ (Supplementary Figure S2B,C). Half-activation potential (V_1/2 act_) values were −8.75 ± 0.54 mV [control] and 9.30 ± 0.86 mV [HOCl-LDL], while activation curve slope factor (k) values were 6.01 ± 0.49 [controls] and 6.13 ± 0.47 [HOCl-LDL]. On the other hand, half-inactivation potential (V_1/2 inact_) and inactivation curve slope factor (k) values were −19.66 ± 1.45 mV and 4.59 ± 0.15 for controls, while values of −21.47 ± 1.04 mV and 5.12 ± 0.33 were found for the HOCl-LDL group, respectively. In line with the qPCR data (Figure 3A), KN93 prevented HOCl-LDL-induced I_Ca,L_ density reduction at membrane potentials of 0 and +10 mV (Figure 3B). The maximum current densities at 0 mV were 7.49 ± 0.67 pA/pF for the HOCl-LDL group and 10.68 ± 0.77 pA/pF for the KN93+HOCl-LDL group.

HOCl-LDL-reduced Kir2.2 channel expression may alter I_K1_, which is a major determinant of the terminal AP repolarization and is crucial for the setting of V_rest_ of atrial and ventricular cardiomyocytes [35,36]. Indeed, HOCl-LDL significantly reduced I_K1_ density (e.g., 0.82 ± 0.12 pA/pF [control] vs. 0.28 ± 0.08 pA/pF [HOCl-LDL] at −40 mV, −27.21 ± 1.29 pA/pF [control] vs. −22.3 ± 1.22 pA/pF [HOCl-LDL] at −130 mV) (Figure 3C). In contrast, LDL treatment (250 µg/mL, 12–16 h) was ineffective (Supplementary Figure S2D). Notably, CaMKII oxidation was found to be the trigger for HOCl-LDL-modulated I_K1_. KN93 pre-treatment significantly preserved I_K1_ density (e.g., 0.28 ± 0.08 pA/pF vs. 0.72 ± 0.12 pA/pF at −40 mV and −22.3 ± 1.22 pA/pF vs. −27.7 ± 1.56 pA/pF at −130 mV for HOCl-LDL and KN93 + HOCl-LDL, respectively; Figure 3C). In parallel, KN93 ameliorated HOCl-LDL-induced APD prolongation (579.93 ± 37.96 ms [HOCl-LDL] vs. 455.58 ± 29.51 ms [KN93+HOCl-LDL], Figure 3D) and maximal upstroke velocity reduction (138.75 ± 17.73 V/s [HOCl-LDL] vs. 260.08 ± 13.07 V/s [KN93+HOCl-LDL], Figure 3E).

Altogether, HOCl-LDL reduced CaV1.2 and Kir2.2 expression, as well as impaired I_Ca,L_ and I_K1_ via CaMKII oxidation. Furthermore, KN93 preserved APD as well as maximal upstroke velocity in HOCl-LDL-incubated cardiomyocytes.

### 3.5. HOCl-LDL Modulates Ca^2+^ Homeostasis and Contractility via CaMKII Oxidation

**Figure 4:**
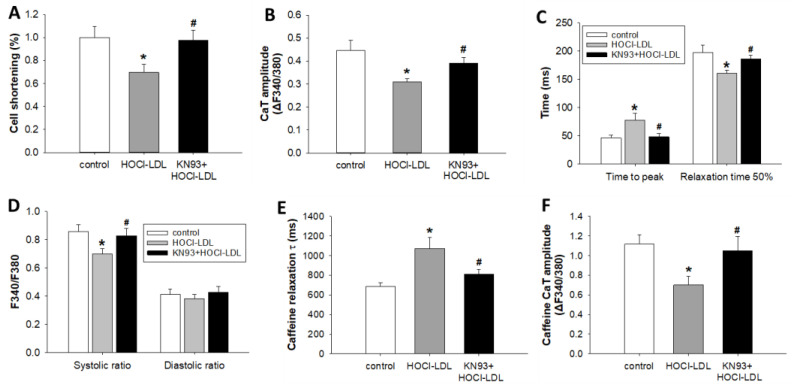
HOCl-LDL-induced CaMKII oxidation modulates cardiomyocyte contractility and Ca^2+^ homeostasis. GPV cardiomyocytes were incubated with HOCl-LDL (oxidant:lipoprotein molar ratio of 200:1, 250 µg/mL, 14 h) in the absence or presence of KN93 (5 µM, added 30 min prior to HOCl-LDL). (**A**) Cell shortening, (**B**) CaT amplitude, (**C**) time to peak and relaxation time (50%), (**D**) CaT systolic and diastolic ratio, (**E**) Caffeine-induced CaT decay τ, and (**F**) Caffeine-induced CaT amplitude were analyzed for cardiomyocytes stimulated at (**A**–**D**) 1 Hz or (**E**,**F**) unstimulated (*n* = 22). Values are expressed as mean ± SEM. (*n*) represents the number of cardiomyocytes. * indicates *p* < 0.05 vs. control, # indicates *p* < 0.05 vs. HOCl-LDL.

CaMKII oxidation and reduced I_Ca,L_ density in HOCl-LDL-incubated cardiomyocytes prompted us to investigate Ca^2+^ homeostasis and contractility of GPV cardiomyocytes. As expected, HOCl-LDL treatment reduced cardiomyocyte shortening (~30%, Figure 4A) and CaT amplitude (~32%, Figure 4B). Moreover, the reduced RyR2 expression (Figure 3A), as well as I_Ca,L_ density (Figure 3B), resulted in a delayed time to peak (~41% reduction, Figure 4C) and lower systolic Ca^2+^ level (~19%, Figure 4D). On the contrary, HOCl-LDL treatment accelerated time to 50% relaxation (~23% increment, Figure 4C), which is corroborated by the elevated SERCA2a expression (Figure 3A). Conversely, diastolic Ca^2+^ level remained unaffected (Figure 4D).

Since NCX1 expression is reduced in HOCl-LDL-incubated cardiomyocytes (Figure 3A), caffeine (a RyR2 agonist) was employed to estimate the contribution of NCX1 in Ca^2+^ extrusion, as described previously [37]. Indeed, decay τ of the caffeine-induced CaT was prolonged (~56%, Figure 4E), indicating impaired NCX1 function in Ca^2+^ extrusion. Surprisingly, we also observed a lower caffeine CaT amplitude (~37%, Figure 4F). This phenomenon indicates a reduced Ca^2+^ content of the sarcoplasmic reticulum (SR). In comparison to HOCl-LDL, incubation with native LDL was ineffective towards Ca^2+^ homeostasis and contractility (data not shown).

Pre-treatment of cardiomyocytes with KN93 prevented the deleterious effects of HOCl-LDL on cell shortening (Figure 4A), CaT amplitude (Figure 4B), time to peak (Figure 4C), relaxation time 50% (Figure 4C), systolic Ca^2+^ level (Figure 4D), caffeine CaT decay τ (Figure 4E), and caffeine CaT amplitude (Figure 4F). These data reveal oxCaMKII-dependent modulation of Ca^2+^ homeostasis and cardiomyocyte contractility in response to HOCl-LDL.

### 3.6. HOCl-LDL Induces Arrhythmia via I_NaL_ Activation

**Figure 5:**
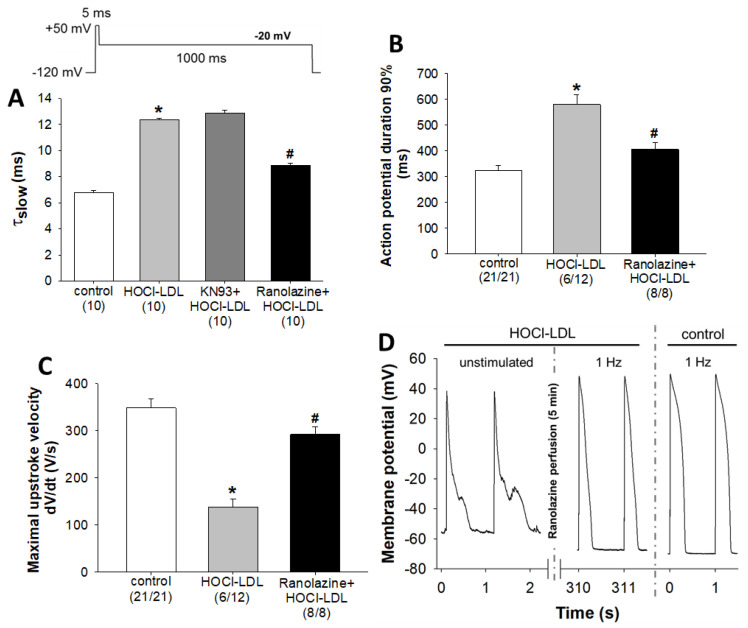
oxCaMKII-independent activation of I_NaL_ by HOCl-LDL. (**A**) Time constant of the slow inactivation phase (τ_slow_) of I_Na_ as a measure of I_NaL_ in GPV cardiomyocytes incubated with HOCl-LDL (oxidant:lipoprotein molar ratio of 200:1, 250 µg/mL, 12–16 h) with or without KN93 (5 µM) or ranolazine (10 µM) added 30 min prior to HOCl-LDL (the voltage clamp protocol is illustrated in the inset). (**B**) APD at 90% repolarization and (**C**) maximal upstroke velocity of GPV cardiomyocytes treated with HOCl-LDL (oxidant:lipoprotein molar ratio of 400:1, 250 µg/mL, 12–16 h, 1 Hz stimulation frequency) with or without ranolazine (10 µM, added 30 min prior to HOCl-LDL). (**D**) Representative AP recordings from a HOCl-LDL (oxidant:lipoprotein molar ratio of 400:1, 250 µg/mL, 12 h)-incubated GPV cardiomyocyte before (0–2 s, unstimulated) and after (310–311 s, stimulated at 1 Hz frequency) superfusion with ranolazine (10 µM, 5 min). For comparative purposes, representative APs of a control cardiomyocyte (0–1 s, stimulated at 1 Hz frequency) are shown. Values are expressed as mean ± SEM. (*n*) represents the number of cardiomyocytes and (n/n) represents the number of myocytes showing stimulated APs/total number of patched myocytes. Cardiomyocytes showing arrhythmic events were excluded from the analysis. * indicates *p* < 0.05 vs. control, # indicates *p* < 0.05 vs. HOCl-LDL.

Although KN93 ameliorated the detrimental effects of HOCl-LDL on I_Ca,L_, I_K1_, ion channel expression, and Ca^2+^ homeostasis, it could not completely block the arrhythmic episodes. Nearly 35% of myocytes showed arrhythmic events in the KN93+HOCl-LDL group compared to 50% of myocytes in the HOCl-LDL group. Moreover, the observed reduction of I_Ca,L_ (Figure 3B) is in contrast to the prolonged APD (Figure 1C). In this regard, we hypothesized that HOCl-LDL-induced superoxide anions and further H_2_O_2_ levels may activate I_NaL_, as this current is known to be activated by H_2_O_2_ and to promote arrhythmic events like EADs and DADs, and APD prolongation [38] (original recordings of I_NaL_ measurements are shown in Supplementary Figure S2E,F). The bi-exponential time course of I_Na_ inactivation was analyzed and the slow inactivation time constant (τ_slow_) was assessed as a measure of I_NaL_ [24]. As shown in Figure 5A, HOCl-LDL increased τ_slow_ in GPV cardiomyocytes (6.79 ± 0.13 ms [control] vs. 12.36 ± 0.10 ms [HOCl-LDL]). Interestingly, HOCl-LDL-induced I_NaL_ was not affected by KN93 pre-treatment (12.36 ± 0.10 ms [HOCl-LDL] vs. 12.88 ± 0.2 ms [KN93+HOCl-LDL], Figure 5A, Supplementary Figure S2E). On the contrary, ranolazine, an anti-arrhythmic drug and a specific blocker of I_NaL_ [39], prevented HOCl-LDL-increased τ_slow_ (12.36 ± 0.10 ms [HOCl-LDL] vs. 8.86 ± 0.15 ms [ranolazine+HOCl-LDL], Figure 5A, Supplementary Figure S2F).

In parallel, an AP parameter analysis showed a reversal of HOCl-LDL-induced APD prolongation (579.93 ± 37.96 ms [HOCl-LDL] vs. 406.12 ± 25.10 ms [ranolazine+HOCl-LDL], Figure 5B) and maximal upstroke velocity reduction (138.75 ± 17.73 V/s [HOCl-LDL] vs. 291.90 ± 15.76 V/s [KN93+HOCl-LDL], Figure 5C). Furthermore, the ratios of stimulated cells to patched cells (given in parantheses in Figure 5B,C) reveal that ranolazine completely abolished HOCl-LDL-induced arrhythmic events, as all myocytes in the ranolazine+HOCl-LDL group could be stimulated for AP measurements compared to 50% of arrhythmic myocytes in the HOCl-LDL group.

As none of the myocytes incubated with HOCl-LDL in the presence of ranolazine showed arrhythmic events, we further examined whether ranolazine could also acutely reverse the arrhythmic events. Therefore, we recorded APs of the HOCl-LDL-treated cardiomyocytes (showing arrhythmias), followed by superfusion with ranolazine (10 µM) for 5 min and further recording of APs (Figure 5D). Representative APs of an unstimulated cardiomyocyte treated with HOCl-LDL exhibited EADs, DADs, and a strongly depolarized unstable diastolic membrane potential of around −54 mV (Figure 5D, left panel). Ranolazine superfusion repolarized and stabilized the diastolic membrane potential to a V_rest_ of approx. −67 mV and APs elicited by an external stimulus at 1 Hz frequency showed no arrhythmic events (Figure 5D, middle panel) and resembled APs of the control cardiomyocytes (Figure 5D, right panel). These data illustrate KN93-independent activation of I_NaL_ in HOCl-LDL-incubated cardiomyocytes. Furthermore, the specific I_NaL_ blocker, ranolazine, eliminates HOCl-LDL-induced arrhythmic events and restores the AP parameters.

## 4. Discussion and Conclusions

Multiple mechanisms have been addressed to understand the impact of MPO on the cardiovascular system. One of the major mechanisms is apparently the modification of LDL by HOCl, which alters protein phosphatases and mitogen-activated protein kinases [40], and increases the atherogenic potential of LDL [10]. Footprints of MPO and HOCl-LDL have originally been detected in human atherosclerotic lesion material [41]. To our knowledge, the present study is the first to provide evidence for the correlation of HOCl-modified epitopes and apoB-100 with cardiomyocytes in the human infarcted myocardium. Previously, mass spectrometry analysis showed that the apoB-100 moiety of in vitro modified LDL by HOCl (oxidant:lipoprotein ratio of 625:1) had similar post-translational modifications as compared to apoB-100 from LDL-particles isolated from patients at high cardiovascular risk and with concomitantly high circulatory MPO levels [42]. On a functional level, MPO^−/−^ mice showed a lesser LV dilation and a better LV function compared to wild-type mice with MI [43]. Moreover, incubation of human ventricular cardiomyocytes with MPO and H_2_O_2_ impaired cardiac contractility [44]. Such electrophysiological impairments support the clinical observation, where high circulatory MPO levels were associated with the higher risk of atrial fibrillation [45]. Furthermore, in a mouse model of myocardial ischemia, MPO promoted arrhythmogenic ventricular remodeling [31].

Numerous preclinical and clinical studies indicate a crucial role of oxidative stress in the development and progression of CVD [2,46], with superoxide anions being the major free radicals in cardiomyocytes and other cell types [2]. Previously, neutrophil-derived HOCl oxidized cardiac myoglobin after acute MI [47]. Additionally, treatment of cardiac myoblasts with HOCl resulted in reduced glutathione levels, altered mitochondrial membrane potential, and necrosis [48]. In the present study, treatment of HL-1 cardiomyocytes with HOCl-LDL elevated the production of superoxide anions, which undergo dismutation to generate H_2_O_2_. Indeed, H_2_O_2_ has a capacity to oxidize isolated, as well as intracellular, CaMKII [33]. The present data show that incubation of cardiomyocytes with HOCl-LDL, MPO-LDL, or H_2_O_2_ resulted in the oxidation of CaMKII at M^281/282^ residues. Furthermore, LOX-1 silencing inhibited HOCl-LDL-induced oxCaMKII expression, revealing a novel LOX-1-mediated signaling cascade that proceeds CaMKII oxidation and further cardiomyocyte dysfunction. Previously, LOX-1 expression was found to be increased in ischemia-reperfusion injury [49], while the administration of an anti-LOX-1 antibody reduced infarct size to 50% in rats undergoing ischemia-reperfusion injury [50].

Most importantly, our results confirm the capacity of HOCl-LDL to induce CaMKII oxidation in the human heart tissue. Additionally, our data showing oxCaMKII expression in the infarcted border region are in line with the previous reports linking oxCaMKII with MI [51]. Upon phosphorylation, CaMKII regulates various cardiac ion channels that contribute to cardiac excitability and contractility [34]; however, the impact of CaMKII oxidation on the cardiac ion channels is not fully understood yet. Increasing evidence reveals that impaired cytosolic Ca^2+^ homeostasis in failing cardiomyocytes is attributed to the dysfunction of one or more Ca^2+^-handling proteins including CaV1.2 and RyR2. Our data show that CaMKII oxidation decreased CaV1.2 expression and reduced I_Ca,L_ density in cardiomyocytes. Similarly, I_Ca,L_ density was reduced in hamster cardiomyocytes treated with HOCl [30]. In contrast, copper-oxidized LDL was reported to increase I_Ca,L_ density in GPV cardiomyocytes [23], as well as in rat ventricular and HEK293-cells [52]. In the latter study, the increase of I_Ca,L_ density is attributed to mitochondrial ROS production mediated by lysophosphatidylcholine. Copper-oxidized LDL has been reported to induce relatively rapid alterations in cellular Ca^2+^ transients via a modification of Ca^2+^ entry through the L-type Ca^2+^ channel in isolated rabbit cardiomyocytes [53] and to affect load-free cell shortening of cardiomyocytes in a PCSK9-dependent manner [54]. However, the use of much higher LDL concentrations and the fact that non-physiological copper-oxidation (in contrast to HOCl-modification [27,28]) leeds to fragmentation of the apoB-100 moiety of the LDL-particle make these studies less comparable.

In the present study, oxCaMKII reduced RyR2 expression, which manifested in delayed CaT time to peak. Previously, digitoxin-induced CaMKII oxidation phosphorylated RyR2 at the S^2814^ residue, resulting in faster CaT time to peak [55]. Our data show that reduced I_Ca,L_ and slower Ca^2+^ release via RyR2 resulted in lower systolic Ca^2+^ levels and impaired cell shortening, where the lower SR Ca^2+^ content may also play a substantial role. Indeed, abnormalities in SR Ca^2+^ homeostasis are the hallmarks of HF and MI; therefore therapeutic approaches targeting Ca^2+^ handling proteins have been proposed [56,57].

HOCl-LDL modulated the Ca^2+^ removal proteins, SERCA2a and NCX1, which contribute to cardiac relaxation. Previously, nitric-oxide-mediated CaMKII oxidation facilitated SR Ca^2+^ reuptake via SERCA2a in cardiomyocytes undergoing adrenergic stimulation [58]. Our results show an oxCaMKII-mediated increase in SERCA2a expression and reduction in CaT relaxation time, which may compensate for the reduced SR Ca^2+^ content. Additionally, the observed reduction of NCX1 expression corroborated reduced Ca^2+^ extrusion via NCX1 during CaT relaxation, a process that may protect cardiomyocytes against further Ca^2+^ loss. In contrast to our findings, Wang and colleagues did not find any change in NCX1 activity in a mouse model of Duchenne muscular dystrophy showing high oxCaMKII levels [59].

The pro-arrhythmic potential of CaMKII has been well established, whereby CaMKII inhibition by various means showed protection against atrial and ventricular arrhythmias. A significant number of studies support a detrimental role of oxidative stress and oxCaMKII in pro-arrhythmic events [60]. Our data demonstrate that HOCl-LDL (i) depolarizes membrane resting potential, (ii) reduces Kir2.2 expression and I_K1_ density via oxCaMKII, and (iii) increases I_NaL_ independent of oxCaMKII. The alterations in current densities cause APD prolongation, which together with the depolarized V_rest_ accounts for the occurrence of arrhythmic events observed in HOCl-LDL-treated cardiomyocytes. Depolarization of V_rest_ reduces Na^+^ current that accounts for the deceleration of AP upstroke velocity [58,59] and may be due to alterations in both, I_K1_ and I_NaL_, since KN93 as well as ranolazine tended to restore V_rest_, although not in a statistically significant manner. Further studies are required to examine the extent to which KN93 and ranolazine can restore V_rest_ in HOCl-LDL-treated myocytes. In line with our data, a chronic CaMKII inhibition in mice resulted in AP shortening via increased I_K1_ density [61], whereas CaMKII overexpression down-regulated Kir2.1 expression and reduced I_K1_ density in rabbit ventricular myocytes [62]. A recent study reported that KN93 may have Ca^2+^-/CaM-related effects independent of CaMKII [63]. However, we followed several lines to corroborate the involvement of oxCaMKII activity in the effects of HOCl-LDL. In accordance with previous studies, KN93 remains a highly effective tool to reduce CaMKII activity and therefore was used as such in the present study. Increased CaMKII activity upon oxidation has been shown in a variety of studies. We robustly demonstrate CaMKII oxidation in HL-1 cardiomyocytes as well as in human intact myocardium (RAA). A typical consequence of oxidative CaMKII activation is increased arrhythmogeneity related to sarcoplasmic reticulum Ca^2+^ leak through CaMKII-dependent post-translational modification of RyR2, which is inhibited by KN93 and also by the unrelated CaMKII-inhibitor AiP [64]. Similar arrhythmias were observed with HOCl-LDL (Figure 1), and are not easily explained by the pattern of altered Ca^2+^-handling protein expression as found in our study (i.e., RyR mRNA decrease and SERCA mRNA increase). Based on these observations, even though we cannot exclude additional non-CaMKII-dependent Ca^2+^/CaM-mediated effects, our data strongly suggest a pivotal role of oxCaMKII-dependent post-translational modification of excitation contraction coupling-proteins in the observed cellular phenotype.

HOCl-LDL treatment triggered I_NaL_, which is the remnant inward Na^+^ current following the peak I_Na_, albeit substantial enough for APD prolongation and EADs. Therefore, I_NaL_ plays a significant pathophysiological role in promoting atrial and ventricular arrhythmias during HF and MI, since it substantially contributes to intracellular Na^+^ overload, which in turn increases intracellular Ca^2+^ levels, possibly causing arrhythmias and diastolic dysfunction [24,65]. A previous study reported the CaMKII-mediated activation of I_NaL_ in ventricular myocytes [24], while a computation analysis suggested oxCaMKII-reduced Na^+^ conduction and I_Na_ density [66]. On the contrary, KN93 was ineffective towards HOCl-LDL-induced I_NaL_ in the present study. Since oxidative stress is one of the major activators of this current, superoxide anion production is the most likely reason for I_NaL_ activation in the HOCl-LDL-treated cardiomyocytes. Previously, H_2_O_2_ treatment of cardiomyocytes resulted in an increased I_NaL_, arrhythmia, and contractile dysfunction that were ameliorated by the Na^+^ channel blocker tetrodotoxin and the clinically used I_NaL_ blocker ranolazine [67]. In our study, the use of ranolazine restored APD 90% and maximal upstroke velocity of Aps, and completely inhibited the occurrence of EADs and DADs in HOCl-LDL-treated cardiomyocytes. In line with this data, another I_NaL_ blocker, GS967, also inhibited EADs, DADs, ventricular tachycardia, and atrial fibrillation in rat hearts [68].

Activated and invading/recruited neutrophils (containing up to 5% MPO of total cell protein content) play potential roles in the initiation and progression of atherosclerosis and other CVDs [9,69] including cardiac damage [3,70]. Thus, MPO has gained significant attention as an oxidative mediator of reperfusion injury, adverse ventricular remodelling, and atrial fibrillation [3]. Neutrophil/MPO-derived HOCl, generated in the heart/myocardium is prone to modify cardiac proteins [47,71].

Our data reveal that the staining pattern of apoB-100 matched to those of HOCl-modified epitopes in the serial sections of LV from MI patients, supporting the evidence that the MPO-H_2_O_2_-Cl^−^ system is active in human infarcted hearts. The modification of LDL via the MPO-H_2_O_2_-Cl^−^ system generates HOCl-LDL, which activated LOX-1 signaling, induced oxidative stress, and oxidized CaMKII in cardiomyocytes. Our data provide a novel mechanism of oxCaMKII-mediated alterations in the expression and function of cardiac ion channels and pumps that trigger arrhythmias and contractile dysfunction. A recent study suggested that the inhibition of cardiac MPO alleviates relaxation defects in cardiomyocytes [72]. As such, the present study suggests a mechanistic insight into the detrimental effects of HOCl that may favour the understanding of cardiac remodeling events in patients with high circulatory MPO levels. Finally, our data may provide a basis for the development of therapeutic strategies against cardiomyocyte dysfunction via oxCaMKII and I_NaL_ inhibition.

## 5. Limitations

The CaMKII inhibitor, KN93, inhibits not only oxCaMKII activity but also pCaMKII activity. However, due to the lack of a specific oxCaMKII inhibitor, we have used KN93, which is widely used to study oxCaMKII signaling in various studies. As KN93 may also bind directly to the Ca^2+^/CaM [63], Ca^2+^/CaM-dependent and non-CaMKII activities might also be involved in altered Ca^2+^ handling. Furthermore, we designed and tested three primer pairs to estimate mRNA expression of GP RyR2 using qPCR; however, none of the primers gave reliable results. Therefore, RyR2 expression is shown only for HL-1 cardiomyocytes and RAAs.

Although HL-1 cardiomyocytes, an immortalized atrial cardiomyocyte cell line, are different compared to primary GP ventricular cardiomyocytes, they were turned out and thus used for time-dependent experiments. The identical pattern of ion channel expression under HOCl-LDL treatment in HL-1 and GPV cardiomyocytes as well as RAAs makes this a reasonable approach.

In addition to the modification of the protein moiety, lipids are also prone to be modified. A potential player generated by the oxidative attack of plasmalogen (an ether-phospholipid present in lipoproteins such as LDL and high-density lipoprotein particles [73,74]) by HOCl, added as a reagent or generated by the MPO-H_2_O_2_-chloride system, is 2-chlorohexadecanal, which is an aldehyde with potent biological effects [75].

## Figures and Tables

**Table 1:** Sequences of the primers used for qPCR. H-human; M-mouse; GP-guinea pig.

Gene	Species (Accession ID)	Forward Primer (5′-3′)	Reverse Primer (5′-3′)
GAPDH	H (NM_002046.7)	Hs_GAPDH_1_SG (Qiagen)	
	M (NM_001289726.1)	Mm_Gapdh_3_SG (Qiagen)	
	GP (NM_001172951.1)	GTGAAGCAGGCATCAGAGGGC	GGCTCAGGTGGGGTCCACTTAC
CaV1.2	H (NM_199460.4)	GAGAACAGCAAGTTTGACTTTGACAA	CGAAGGTGGAGACGGTGAA
	M (NM_009781.4)	Mm_Cacna1c_1_SG (Qiagen)	
	GP (NM_001172923.1)	GCGGACACAGAGGTGAGGGG	GTGGGGATGTGCTCAGGGGC
NCX1	H (NM_001112800.4)	CTGGTGGAGATGAGTGAGAAGA	GGTTGGCCAAACAGGTATTTTC
	M (NM_011406.3)	CCCTGTTGTTGAATGAGCTTGGTGG	TGCTGGTCAGTGGCTGCTTGT
	GP (NM_001173019.1)	TCGCCCTCCACTGCCACTGT	TGACCTCCATGATGCCAATGCTCT
SERCA2a	H (NM_001681.4)	CCGCAACTACCTGGAACCTG	CACGCAACCGAACACCCTTA
	M (NM_001110140.3)	CACGTGCCTGGTGGAGAAGATGA	CCGGCTTGGCTTGTTTGGGG
	GP (XR_001199631.2)	AGGTGCTGGGCCACTTCGGT	TTCAGCCGGTAACTCGTTGGAGC
RyR2	H (NM_001035.3)	GGCGAAGACGAGATCCAGTT	CTTTGTGGATGGTTGCGGTG
	M (NM_023868.2)	CCATGGCTGATGCGGGCGAA	GCAGGGCCCGTACTGACAGG
Kir2.1	H (NM_000891.3)	TTCAGTCACAATGCCGTGATT	GCTTTTCCGAAGATTGCCCA
	GP (NM_001172975.1)	TGTGTCCATGCTCCCGTGCC	TGCTGAGGACGCCAGTGCTT
Kir2.2	H (NM_021012.5)	GCCCACTCAGCACCATTACA	CCTCCTCCGATGACACGATG
	M (NM_010603.6)	GAGTCTGTGCCCACTGTGCCTG	TTGGGGTACTCAGACGCCGGG
	GP (NM_001173037.1)	ACGCAGACCACCATCGGCTAC	GCCACCACCATGAAGACAGCCAC
Kir2.3	H (NM_152868.3)	ACCTCAACGTGGGCTATGAC	CGTCGATCTCGTGGACAATGAT
	GP (NM_001172710.1)	GTCTTCCCAGGTGACACGCCG	TCTTGACGAAGCGGTTGCGG
SR-A1	M (NM_031195.2)	TGAACGAGAGGATGCTGACTG	TGTCATTGAACGTGCGTCAAA
SR-B1	M (NM_016741.2)	TTTGGAGTGGTAGTAAAAAGGGC	TGACATCAGGGACTCAGAGTAG
CD36	M (NM_001159558.1)	AGATGACGTGGCAAAGAACAG	CCTTGGCTAGATAACGAACTCTG
LOX-1	M (NM_138648.2)	CAAGATGAAGCCTGCGAATGA	ACCTGGCGTAATTGTGTCCAC

## Data Availability

The data presented in this study are available in the article and supplementary material.

## Supplementary Materials

The following are available online at https://www.mdpi.com/article/10.3390/antiox11010025/s1, Figures S1–S3.
