# Supplementary material for: Hypochlorite-Modified LDL Induces Arrhythmia and Contractile Dysfunction in Cardiomyocytes
Source: Antioxidants (Basel). 2021 Dec 23;11(1):25. doi: 10.3390/antiox11010025 (PMC8772905; doi:10.3390/antiox11010025)
Supplement: Supplementary file 1 [file antioxidants-11-00025-s001.zip › antioxidants-1511223-supplementary.pdf]

antioxidants

MDPI

Supplementary Material: Hypochlorite-Modified LDL Induces Arrhythmia and Contractile Dysfunction in Cardiomyocytes

Chintan N. Koyani [1,2,†], Susanne Scheruebel [3,†], Ge Jin [2,4], Ewald Kolesnik [2], Klaus Zorn-Pauly [3], Heinrich Mächler [5], Gerald Hoefler [6], Dirk von Lewinski [2], Frank R. Heinzel [7], Brigitte Pelzmann [3,*] and Ernst Malle [1,*]

1 Division of Molecular Biology and Biochemistry, Gottfried Schatz Research Center, Medical University of Graz, Graz 8010, Austria; chini.koyani@novatium.at (C.N.K.)
2 Department of Internal Medicine, Division of Cardiology, Medical University of Graz, Graz 8036, Austria; ewald.kolesnik@medunigraz.at (E.K.); dirk.von-lewinski@medunigraz.at (D.v.L.)
3 Division of Biophysics, Gottfried Schatz Research Center, Medical University of Graz, Graz 8010, Austria; susanne.scheruebel@medunigraz.at (S.S.); klaus.zornpauly@medunigraz.at (K.Z.-P.)
4 The 2nd Affiliated Hospital and Yuying Children's Hospital of Wenzhou Medical University, 325000 Wenzhou, China; gemason@wzhealth.com (G.J.)
5 Department of Surgery, Division of Cardiac Surgery, Medical University of Graz, Graz 8036, Austria; heinrich.maechler@medunigraz.at
6 Diagnostic and Research Center for Molecular BioMedicine, Diagnostic and Research Institute of Pathology, Medical University of Graz, Graz 8010, Austria; gerald.hoefler@medunigraz.at
7 Department of Internal Medicine and Cardiology, Charité-Universitätsmedizin Berlin and DZHK (German Centre for Cardiovascular Research), partner site Berlin, Campus Virchow-Klinikum, 13353 Berlin, Germany; Frank.heinzel@charite.de (F.R.H.)
* Correspondence: brigitte.pelzmann@medunigraz.at (B.P.); ernst.malle@medunigraz.at or ernst.malle55@gmail.com (E.M.)
† These authors equally contributed to this work as first authors.

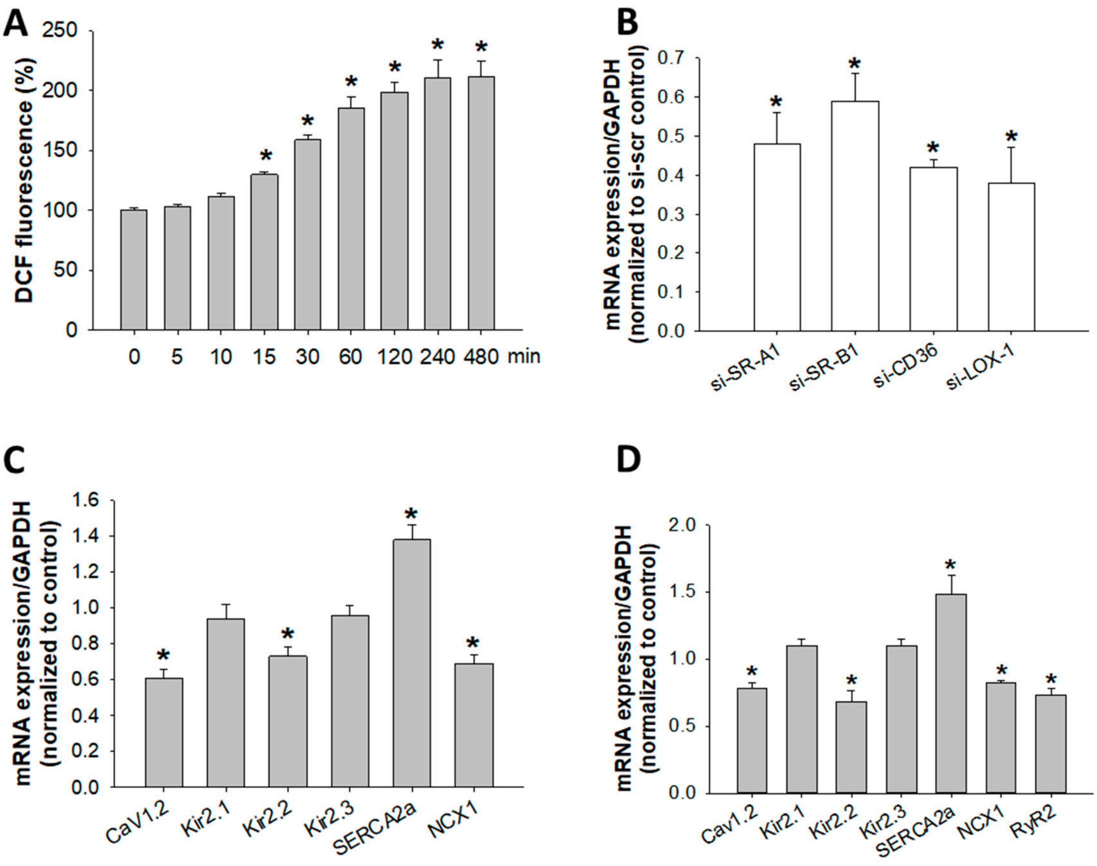

Figure S1. (**A**) Time-dependency of ROS/RNS production, as reflected by DCF fluorescence intensities, in HL-1 cardiomyocytes treated with HOCl-LDL (oxidant:lipoprotein molar ratio of 200:1, 250 µg/mL) for indicated time periods (n = 6). (**B**) Efficiency of indicated siRNA to reduce

mRNA expression of the respective scavenger receptor in comparison to si-scr transfection of HL-1 cardiomyocytes (n = 6). The mRNA expression of indicated ion channels/pumps in (**C**) GPV cardiomyocytes (n = 6) and (**D**) human RAAs (n = 3) incubated with HOCl-LDL (oxidant:lipoprotein molar ratio of 200:1, 250 µg/ml) for (**C**) 12 h and (**D**) 8 h. Values are expressed as mean±SEM. (n) represents the number of experiments. $^{*}p < 0.05$ vs. control.

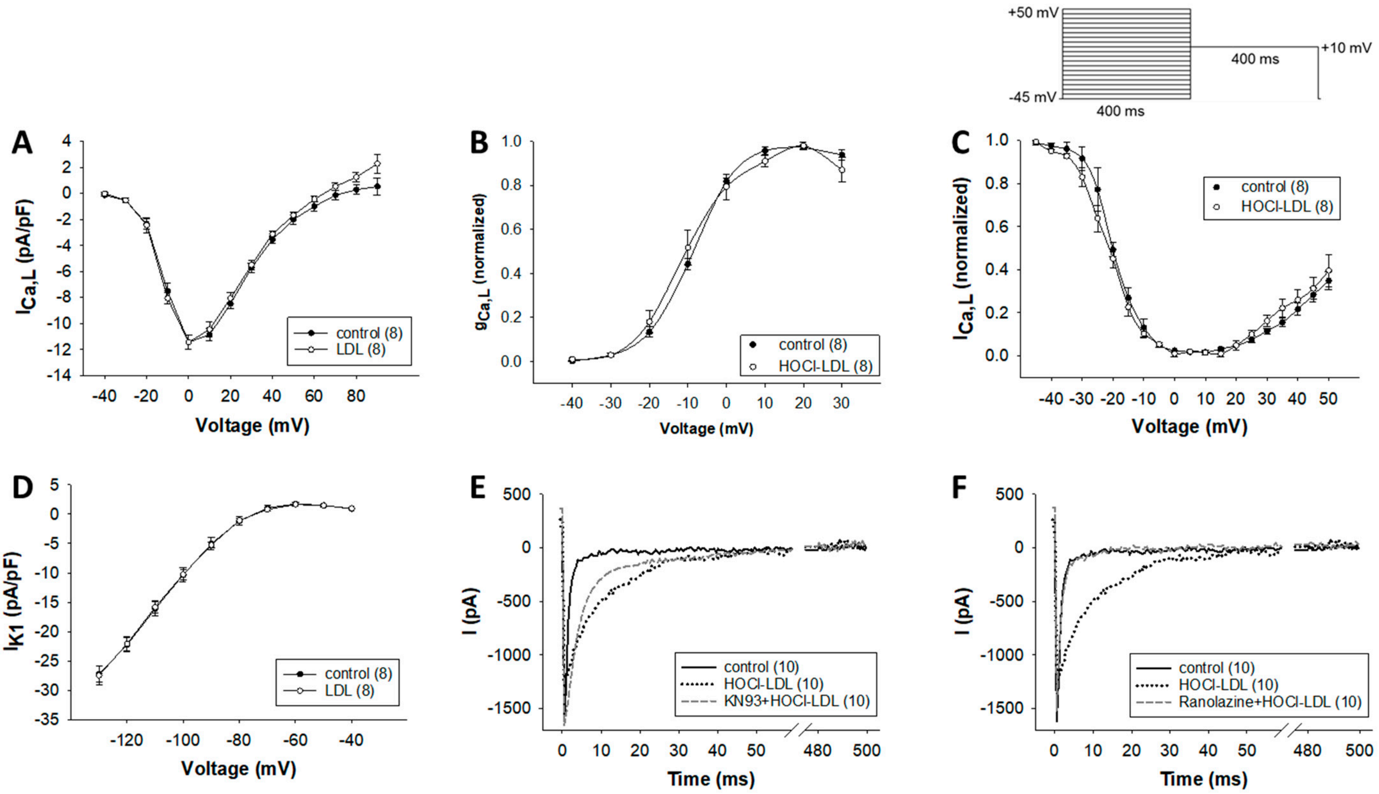

Figure S2. (**A**) $I_{Ca,L}$ and (**D**) I_{K1} density of GPV cardiomyocytes incubated with native LDL (250 µg/ml, 12-16 h). Steady-state (**B**) activation and (**C**) inactivation of $I_{Ca,L}$ in response to HOCl-LDL (oxidant:lipoprotein molar ratio of 200:1, 250 µg/mL, 12-16 h) treatment. The voltage clamp protocol for the steady-state $I_{Ca,L}$ inactivation measurements is illustrated in the respective inset. (**E**,**F**) Representative current traces of I_{NaL} measurements using GPV cardiomyocytes incubated with HOCl-LDL (oxidant:lipoprotein molar ratio of 200:1, 250 µg/mL, 12-16 h) with or without either (**E**) KN93 (5 µM) or (**F**) ranolazine (10 µM) that were added 30 min prior to HOCl-LDL. Values are expressed as mean±SEM. (n) represents the number of cardiomyocytes.

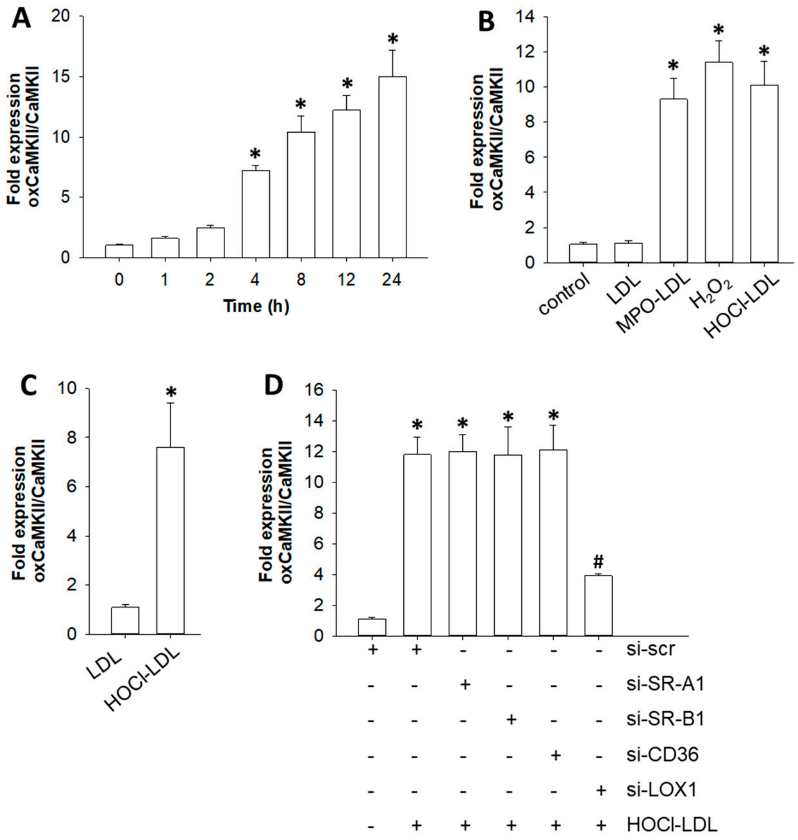

Figure S3. Densitometric evaluation of Western blots shown in (**A**) Figure 2B (n = 6), (**B**) Figure 2C (n = 6), (**C**) Figure 2D (n = 3) and (**D**) Figure 2F (n = 6). Values are expressed as mean±SEM. (n) represents the number of experiments. *$p < 0.05$ vs. control/LDL, #$p < 0.05$ vs. HOCl-LDL.
